# Bioanalytical Assay Strategies and Considerations for Measuring Cellular Kinetics

**DOI:** 10.3390/ijms24010695

**Published:** 2022-12-31

**Authors:** Amanda Hays, Jennifer Durham, Bryan Gullick, Nathan Rudemiller, Thomas Schneider

**Affiliations:** BioAgilytix Labs, 2300 Englert Drive, Durham, NC 27713, USA

**Keywords:** pharmacokinetics, PK, cellular kinetics, CAR-T, cell therapy

## Abstract

A vast evolution of drug modalities has occurred over the last several decades. Novel modalities such as cell and gene therapies have proven to be efficacious for numerous clinical indications–primarily in rare disease and immune oncology. Because of this success, drug developers are heavily investing in these novel modalities. Given the complexity of these therapeutics, a variety of bioanalytical techniques are employed to fully characterize the pharmacokinetics of these therapies in clinical studies. Industry trends indicate that quantitative PCR (qPCR) and multiparameter flow cytometry are both valuable in determining the pharmacokinetics, i.e. cellular kinetics, of cell therapies. This manuscript will evaluate the pros and cons of both techniques and highlight regulatory guidance on assays for measuring cellular kinetics. Moreover, common considerations when developing these assays will be addressed.

## 1. Introduction

Drug development has been evolving dramatically over the past several decades to counter the unmet challenge of previously untreatable diseases. Novel and refined technologies have propelled drug developers beyond small molecule therapeutics and simple biologics (e.g., monoclonal antibodies) into highly complex, large-molecule drugs. Of these complex modalities, the class of cell and gene therapies has progressed exponentially over the last several years, resulting in successful regulatory approvals of several cell and gene therapies for various treatment indications. One such indication is immune oncology; the interplay of the immune system and diseases such as cancer or autoimmunity has led to many breakthroughs in immunotherapy. Additionally, advancements in gene editing techniques and transgene delivery systems are leading to breakthroughs in patient care. Various gene and cell therapy programs have shown great promise for treatment of a multitude of diseases including those in rare disease and difficult to treat cancers.

Gene therapy is the introduction of genetic material into a patient for treatment of a disease. The transfer of genetic material or method of delivery is commonly achieved via viral (e.g., adenovirus, lentivirus, AAV (adeno-associated virus) and non-viral vectors (e.g., lipid nanoparticles, liposomes, polymers, and peptides). Regardless of delivery method, gene therapies can be classified as in vivo or ex vivo. In vivo gene therapies are administered directly to the patient. whereas ex vivo gene therapies entail harvesting patient’s cells for genetic modification outside of the patient with a therapeutic transgene, and then returning the cells to the patient [1,2]. Ex vivo gene therapy can be further classified as adoptive cell therapies that are differentiated into subclasses based on their mechanism of action. Of these, tumor-infiltrating lymphocytes (TILs), T-cell receptor (TCR) therapies, and chimeric antigen receptor (CAR) modified immune cells are of current interest for drug developers. Throughout this review, CAR cell therapy is often referenced. However, the principles applied to measuring cellular kinetics in CAR cell therapy may also be applied to other cell therapies that express unique transgenes.

Although extremely attractive and potentially opportunistic, the development of CAR-based therapeutics is not without challenges that have been summarized in several recent reviews [3,4,5]. An additional degree of complexity of CAR-based therapeutics is the source of cells being delivered to the patient. The cells can be autologous, i.e., derived from the patient’s own cells, or allogeneic, wherein the cells are derived from a source other than the patient. While autologous cell therapies are considered to be safer, significant time in the preparation of these modalities can lead to complications for the sick patient. In contrast, allogeneic cell therapies can be readily available, often referred to as “off-the-shelf” therapeutic products. While allogeneic sources have great clinical potential, significant concerns remain. Namely, rejection in the form of graft versus host disease (GvHD) or other unwanted immune reactions to foreign cells threatens patient safety and cell therapy efficacy [6].

Despite challenges, these therapies have shown remarkable effectiveness in treating different types of blood cancers and have led to the FDA (Food and Drug Administration) approval of several cell therapies to-date, including Kymriah^®^ (tisagenlecleucel) for acute lymphoblastic leukemia [7], Tecartus™ (brexucabtagene autoleucel) for mantle cell lymphoma, Yescarta^®^ (axicabtagene ciloleucel) for B-cell lymphoma and Bryanzi (lisocabtagene maraleucel) for relapsed and refractory large B-cell lymphomas, with all four therapies being autologous CAR-T cells that target CD19 on the surface of B cells [8,9]. The most recent cell therapy approval was for Carvykt (ciltacabtagene autoleucel), an autologous BCMA-directed CAR-T therapy for the treatment of relapsed and refractory multiple myeloma [10]. The approval of these cell therapies is likely only the beginning. The cell-therapy landscape continues to evolve with refinement of CAR constructs, targeting a variety of disease-related antigens, and harnessing other immune cells to expand into solid tumor oncology and autoimmune disorders [11].

Cell therapies are “living cells.” Therefore, they exhibit a unique pharmacokinetic (PK) profile termed cellular kinetics. Cellular kinetic profiles, specifically for CAR-T therapeutics, appear to have multiphasic parameters that include distribution, expansion, contraction, and persistence phases. Moreover, with the characteristic of rapid in vivo expansion, cell therapies can persist up to one year or longer in patients. Therefore, unlike typical small or large molecule therapies, cellular therapies do not show a strong relationship between dose and exposure. Thus, it is imperative to effectively strategize bioanalytical sampling for these studies. The cellular kinetics of the first approved CAR-T cell therapy, Kymriah, were measured and described by Mueller et al. [12], showing the expansion and persistence of the circulating cell therapy in clinical samples.

Cellular kinetics can be quantified using multiple bioanalytical techniques. Although correlative, the different techniques may not always produce concordant data sets. Thus, it is imperative to understand the platform being used, know what is being quantified, and how to compare and interpret the data. Current industry trends utilize two main techniques for resolving cellular kinetics–quantitative polymerase chain reaction (qPCR) and flow cytometry.

**Figure 1:**
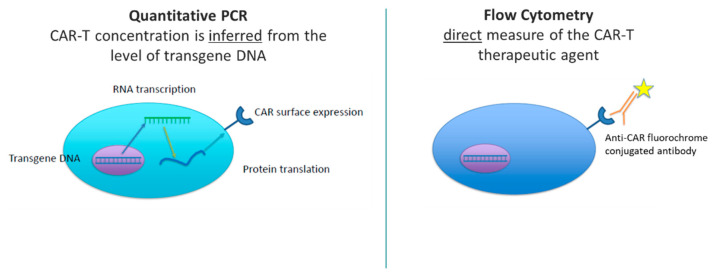
Schematic of CAR-T Detection by qPCR and Flow Cytometry.

With CAR-T cell therapy or other cell therapies, an engineered transgene that encodes the CAR or cell surface therapeutic is introduced in the form of RNA. The RNA is reverse-transcribed into DNA, which is integrated into the genome of the patient’s cells to express the CAR protein at the cell surface. qPCR measures cell concentration through detection of integrated transgene copies per cell. These data are then extrapolated or inferred as the number of circulating CAR-T cells. Whereas flow cytometry provides a direct measure of the cell therapy via antibody-specific fluorescent labeling of the engineered cell surface molecule (e.g., the CAR). The difference in detection of CAR-T therapy between qPCR and flow cytometry is illustrated in Figure 1. Both platforms are valuable in measuring cellular kinetics, and both platforms have advantages and disadvantages. qPCR and flow cytometry will be discussed in the context of measuring cellular kinetics, and common considerations when developing and validating these assays will be addressed.

## 2. Quantitative PCR

From its invention in the early 1980s using oligonucleotide primers, dNTPs, and Taq polymerase for DNA amplification [13], the qPCR workflow has evolved and been used in various applications in food safety, agriculture, diagnostics, and drug development. qPCR is a technique used to amplify and quantify DNA. This versatile platform has many advantages. qPCR assays are rapid, highly specific, robust, and provide exceptional target sensitivity. qPCR remains the ‘gold standard’ for nucleic acid quantification. Moreover, for cellular kinetics, qPCR is the standard for measuring transgene DNA. Reverse transcription qPCR (RT-qPCR) entails transcribing RNA into complementary DNA (cDNA) and using the cDNA as a template for the PCR reaction. RT-qPCR applications are not typically employed in cellular kinetics and are out of scope for this manuscript.

Another molecular platform undergoing significant adoption is digital PCR (dPCR) technology. The concept of dPCR was first described by Sykes et al. wherein the absolute quantification of nucleic acids was achievable through end-point PCR and Poisson statistics [14]. dPCR’s hallmark capability is absolute quantification of nucleic acid concentration in a sample, thereby eliminating the need for a standard curve. The dPCR workflow is summarized in the following sequential steps: sample preparation, partitioning, amplification, and detection. Numerous commercial dPCR platforms have been developed over the years utilizing differing partitioning techniques, though droplet digital PCR (ddPCR) technology is widely used in cell therapy bioanalysis. For dPCR, the sample is prepared just as it would be prepared for traditional qPCR. Then, in the case of ddPCR specifically, the sample is partitioned into uniform droplets that have a few or no target sequences. After the droplets are generated, PCR is used to amplify the sequences. The droplets are then passed through a droplet reader that separates them into positive or negative droplets based on the amount of fluorescence that is above or below a threshold; hence the ‘digital’ aspect; droplets are either fluorescent or they are not. Finally, to calculate the concentration of target in the sample, the technology uses the ratio of positive to total droplets and Poisson distribution statistics [15]. In recent industry trends, both qPCR and various dPCR platforms have been heavily utilized in determining cellular kinetics to determine the number of cells that make up the cellular therapy pharmacokinetic profile. Previous efforts have compared both platforms and advantages of each [16]. One of the most cited advantages of using dPCR over qPCR is the mere fact that a standard curve is not required for absolute quantification of copy numbers. Another attractive reason for developing a dPCR assay is the minimal susceptibility to matrix interferents and PCR inhibitors. Both qPCR and dPCR have comparable sensitivity capabilities. However, an advantage of the dPCR system is superior precision for rare events and at lower copy numbers. Therefore, dPCR has been slightly preferred for the reason of requiring adequate precision at lower sensitivity to detect as low a number of cells as possible for characterizing persistence of cell therapy products. Another advantage of qPCR is the high-throughput capability of the platform, especially with 384-well plate formats. This advantage lends favor for deploying this platform for assays that are used to support global or multi-site clinical trials. Lastly, the cost of qPCR reagents and consumables are more affordable than the often proprietary reagents for dPCR platforms.

### Method Considerations

Collectively, whether using qPCR or dPCR, molecular assays are one of the primary methods for measuring cellular kinetics. To that end, these assays measure the concentration of the therapeutic from the level of transgene DNA in matrix (e.g., peripheral blood, bone marrow, etc.). With cellular kinetics, the primary advantage of qPCR assays over flow cytometry is superior sensitivity. Although not a direct measure of the CAR, qPCR remains the more sensitive method [17] and is reported as transgene copies/µg of DNA or vector copies/mL of blood.

Considerations for developing and validating these assays have been extensively described in recent publications. In general, a qPCR/dPCR method is developed for assessing two targets; the first is designed to detect the CAR transgene DNA and a second that measures a reference gene DNA used to normalize the transgene copy number within each sample. Vector copy number (VCN) assessment measures presence of the CAR vector and CAR cells by determining the average vector copies per genome by using the CAR copy number and genome copies together. In their publication, Yang and Doddareddy [18] summarize recommendations and best practices for developing cellular kinetics assays for CAR therapies via qPCR. Notably, reliability of the VCN can be highly dependent on the specificity of the primers and probes to the target [19], as the technology can slightly overestimate the number of CAR cells in a sample. Universal qPCR assays to detect CAR-T cells in peripheral blood have been described [20]. Such methods can be employed to monitor cellular kinetics for CAR based therapies.

Like all bioanalytical assays, the quality of the sample preparation dictates the quality of results. When developing qPCR for cellular kinetics assays, the nucleic acid extraction method is critical. Different DNA extraction methods (e.g., silica-based versus magnetic bead-based, manual versus automated extraction) can result in varying nucleic acid yields. Thus, the DNA extraction method requires optimization to yield sufficient nucleic acid recovery and highest purity. Another prominently debated topic concerns the use of dPCR and determining the “nominal” concentration of reference material. While the use of absolute quantification is advantageous to avoid the requirement of preparing a standard curve containing known spike concentrations of a reference calibrator, challenges arise in determining which method of concentration determination will provide the most accurate value of the starting calibrator material. For example, when nucleic acid concentration is determined by a default method such as UV absorption, an overestimation of the actual concentration may occur due to the presence of contaminants such as salts, organic solvents, or detergents. Another drawback of using dPCR for cellular kinetics assays is the limited dynamic range. The upper limit of detection is limited by the saturation of positive droplets [21]. Therefore, when using a reference gene, the maximum amount of genomic DNA (gDNA) input in the reaction must be considered to ensure reference gene copies are within the dynamic range of detection.

The reportable units for cellular kinetics assays must also be considered. The common regulatory reportable unit is copies/µg of gDNA for transgene detection. Although useful, copies/µg of gDNA may not be ideal for cellular kinetics. Namely, it may underestimate the cell therapy expansion phase in the kinetics profile [22]. Perhaps a more meaningful reportable unit could be “copies/µL of blood or µg of tissue.” These examples are more pharmacologically meaningful units that characterize expansion of the transgene in vivo. Additionally, by using the pharmacologically relevant unit, a more accurate determination that accounts for dramatic changes in blood gDNA levels after lymphocyte-depleting chemotherapy and rapid expansion of cells is a better representation for cellular kinetics. Moreover, lymphodepletion, a procedure often associated with cell therapies, presents a challenge in measuring cellular kinetics. Samples could lack reference gene expression due to low cell numbers and in turn prohibit reporting the transgene concentration. In this case, reporting the concentration of transgene or target per volume rather than target per µg DNA as a reportable could provide informative data rather than not reporting a result at all or reporting an ‘insufficient material’, ‘BLQ’ or ‘not evaluable’ result.

## 3. Flow Cytometry

Unlike molecular assays, flow cytometry provides a direct measure and characterization of the cell therapy product. As mentioned, the main advantage of using qPCR as a measure of exposure to the cell therapy is the superior sensitivity. However, the presence of the therapeutic transgene is not a means to assess the intact, viable cell therapy in the patient. Multiparameter flow cytometry can resolve viable cell therapy subsets, thus elucidating the phenotype of the cell therapy. Therefore, flow cytometry can provide quantitation while also revealing the physiological state of the cell therapy. In the case of CAR T cells, probing for lineage and effector markers provided significant insight into the phenotypic composition of CAR T cells within cancer patients, showing elevated percentages of effector memory T cells within the CAR population compared to the host counterpart [23].

Multiparameter flow cytometry also provides the ability to simultaneously measure a variety of bioanalytical targets on host immune cells in addition to the cell therapy within a single assay. The ability to measure how the host system responds to the cell therapy adds value in understanding safety and efficacy of the treatment. This is especially useful, given the ability to obtain rich data sets with a small volume of sample.

Flow cytometry technology is continually advancing. Conventional flow cytometers can now measure 20+ markers in a single sample and are invaluable in multiparameter flow cytometry [24]. The next generation flow cytometry platforms, such as mass and spectral cytometry, permit the user the capability to develop panels with 50+ markers, rendering the possibilities endless for developing remarkable panels to resolve cellular kinetics, immune response, cytokine measurement, pharmacodynamic (PD) biomarkers, and others in a single sample.

### Method Considerations

With the extraordinary ability to develop high-parameter readouts comes the responsibility of understanding complex panel design and data analysis. Large panels developed to simultaneously measure 10+ markers require much planning. The basics of sound multiparameter panel design are readily available [25]. Some of the basic suggestions include reserving detectors with the least fluorescent spillover for critical markers and using dim fluorochromes with high-expressing markers. In addition, careful compensation is paramount for informative data in flow cytometry, and careful understanding of the platform and skills for proper compensation requires experienced scientists.

Cellular kinetics is a crucial dataset in support of clinical programs. The flow cytometric assay to measure cellular kinetics will likely require validation. Therefore, prudence in panel design is suggested. Namely, the panel should be focused and not include extraneous markers that would otherwise be reserved for exploratory assays. The number of markers to include in the panel depends on the cytometer capabilities (i.e., number of detectors, detector resolution, etc.) and the experience of the operator. To help facilitate data analysis and appropriate gating strategies, employing isotype controls and FMOs (fluorescence minus one) within the panel mitigates improper gating and non-specific staining within the sample.

Another complicating factor in assay development is often the inability to procure disease matrix. The preferred sample matrix is whole blood, which must be tested within hours or days following collection depending on the stability of the cell therapy. The stability should be determined prior to clinical samples testing. Whole blood allows the cellular kinetic data to be presented as a concentration (e.g., cells/mL blood) or % of population. Peripheral blood mononuclear cells (PBMCs) may also be used as the sample matrix, but the ability to express the data as a concentration is lost, and % of population must be used. Whenever possible, disease matrix should be tested during qualification/validation. Cellular kinetic panels are typically developed using normal matrix. However, certain markers of interest are differentially expressed in disease state samples. Thus, ex vivo perturbation to cells may be necessary to elicit cellular phenotypes representative of disease-state samples.

Additionally, as described in Lang et al. [26], proper bioanalytical reagents for the assay such as antibodies and positive controls are critical. To specifically detect the cell therapy in matrix, antibodies, or other specific detection reagents for the unique, cell-surface biotherapeutic (CAR, TCR, etc.) are needed. In the case of CAR-based cell therapies, antibodies are often used that target the antigen-recognizing scFv (single chain variable fragment) portion of the CAR. Sometimes these antibodies are commercially available, or they are anti-idiotype antibodies proprietary to the pharmaceutical company generating the cell therapy. If a specific detection reagent is not available, generic detection methods have been used. For example, protein L binds to scFv and may be a suitable reagent to detect CAR [27]. In other cases, Histidine (His), FLAG or similar tags have been incorporated into the CAR for detection. The availability of commercial reagents allows for ease of access to reagents for method development and even could provide a control over lot-to-lot differences seen from in-house uncontrolled reagent batch manufacturing. For assay positive controls, transfected cell lines or transduced normal donor T cells can be used, with advantages and drawbacks for each. These positive control cells are spiked into sample matrix to mimic a patient sample.

As mentioned previously, a major drawback of multiparameter flow cytometry as a tool to measure cellular kinetics is the fact that it is not a very sensitive measure, as compared to qPCR. Another limitation is the lack of reference standard/calibration curve to provide absolute quantitation. The drug product is unavailable as a bioanalytical reagent. Therefore, quasi-quantitation of the cell therapy is achieved via enumeration reagents such as fluorescent counting beads or volumetric syringes within the flow cytometer. Cellular kinetics can also be expressed as % of population, a common flow cytometric readout. Notwithstanding these limitations, the quantification using flow cytometry is a direct measure of the cell therapy. Moreover, a high-parameter panel can provide additional insight into the phenotype of the cell therapy. It has been shown that increasing the grade of reagents in a flow cytometry assay can increase the robustness of the assay and provide greater sensitivity as demonstrated by Sarikonda et al. [28]. They showed an increase in sensitivity for detection of 0.01% of CAR-T cells within the total CD3+ compartment in second generation trials as compared to 1.0% in first generation trials when deploying a higher affinity anti-CAR detection agent.

## 4. Regulatory Considerations

One of the largest concerns to date for developing and validating qPCR and flow cytometry assays in support of bioanalytical assays, including cellular kinetics, is the lack of regulatory guidance on how to validate these assays. The FDA, EMA (European Medicine Agency) and ICH (International Council for Harmonization) M10 bioanalytical method validation guidance documents [29,30,31] address validation of pharmacokinetic methods for traditional small molecule therapeutics and large molecule biologics with a focus on chromatography and ligand binding assays. These platforms cannot effectively characterize the PK profile of cell therapies. The platforms necessary to measure cellular kinetics, qPCR, and flow cytometry, are not addressed in the aforementioned guidance documents.

In early years, the 2009 MIQE guidelines [32] that describe the minimal information required for publication of quantitative Real Time PCR experiments were heavily relied upon for development and characterization of qPCR assays in regulated bioanalysis. Since then, several recent publications have described recommendations and best practices for developing and validating qPCR assays in bioanalysis, especially for cell and gene therapies. Recent publications describe best-practices and working recommendations for the purpose of facilitating future regulatory guidance and to initiate discussion on best practices in the industry of regulated bioanalysis [16,33]. Certain performance characteristics have been deemed necessary for validating and properly characterizing these assays. Ultimately, the recommendation is to provide scientific evidence that the method is suitable for its intended purpose and to apply suitable acceptance criteria based on the context of use of the assay [34].

Like qPCR, there are no regulatory guidelines from the health authorities for the validation of flow cytometry assays in support of cellular kinetics assays. The CLSI (Clinical and Laboratory Standards Institute) released the H62 guidance on validation of assays performed by flow cytometry [35] that addresses the uniqueness of this technology since not addressed by the standard FDA BMV or ICH M10 guidance [29,30,31] that is applicable to ligand binding and chromatographic assays.

## 5. Discussion

Despite the differences in both platforms and pros and cons associated with each as listed in Table 1, the body of literature largely supports general concordance of study data between both platforms. In Mueller et al. [12], the authors demonstrate concordance of the kinetics of CTL019 by measuring circulating CD3+CAR+ cells by qPCR and flow cytometry. In Sugimoto et al. [36], the authors demonstrate concordance of flow cytometry and qPCR data by correlation of transgene level and CAR-T expression in mice. Another publication demonstrated comparable kinetics profile with a slightly increased sensitivity for persistence of tisagenlecleucel by qPCR (693 days) as compared to persistence detection by flow cytometry (554 days) [37]. Another data set in Sarikonda et al. demonstrates near perfect correlation using Spearman’s method between CART19+ CD3+ cells analyzed by flow cytometry and CART19 copies/cell analyzed by qPCR [28]. With multiple approaches able to resolve kinetics of a cellular therapeutic, it is important to understand how to integrate these bioanalytical data sets into the larger picture of drug development.

CAR-T therapies have certainly paved the way and opened the door for immunotherapies that harness other immune cells like Natural Killer (NK) cells [38] and macrophages [39] among others. Thus, it is important to continue to understand the challenges and limitations learned with development and approval of CAR-T therapies to continue to resolve best bioanalytical strategies for resolving cellular kinetics.

## 6. Conclusions

As therapeutic modalities increase in complexity, so do the measures needed to quantitate and characterize them. These new challenges also provide exciting opportunities to set the proper precedent for measures that add value to regulated studies. The FDA, EMA, and ICH M10 guidance documents [29,30,31] are written for pharmacokinetic assays that utilize ligand binding and chromatographic assays., With the continually evolving and growing gene and cell therapy field, it is evident that scientists should continue to critically consider what quantitative measurements relate specifically to their drug program. These measurements will likely not be generated using legacy platforms such as chromatography and ligand binding assays. qPCR and flow cytometry are two platforms that have different advantages for quantifying cellular kinetics and will continue to provide valuable data to support what is to come in the cell therapy realm.

With the approval of several cell therapies in recent years and several other cell therapy strategies on the horizon, continued discussion amongst scientists on best practices for resolving cellular kinetics will facilitate the establishment of guidelines on assays to use for regulatory submissions.

## Figures and Tables

**Table 1:** Comparison of qPCR and Flow Cytometry Platforms for Cellular Kinetics.

	Quantitative PCR (qPCR)	Multiparameter Flow Cytometry
Ease of workflow	++	+
Sensitivity	+++	+
Precision of rare events	+++	+
Enumeration of cells	+++	++
Characterization of cellular phenotype	+/-	+++
Ability to characterize all non-functional phases of cellular therapy pharmacokinetics:		
Distribution	++	+
Expansion	++	+
Contraction	++	++
Persistence	++	+
Complexity of required reagents and controls	+	++
Complexity of data analysis	+	+++
Compatibility for multi-site study support and global deployment	++	+

## Data Availability

Not applicable.
